# Cervical cancer screening history prior to a diagnosis of cervical cancer in Danish women aged 60 years and older—A national cohort study

**DOI:** 10.1002/cam4.1926

**Published:** 2019-01-01

**Authors:** Anne Hammer, Vibeke Soegaard, Rikke D. Maimburg, Jan Blaakaer

**Affiliations:** ^1^ Department of Obstetrics and Gynecology Aarhus University Hospital Aarhus Denmark; ^2^ Department of Clinical Medicine Aarhus University Aarhus Denmark; ^3^ Department of Orthopaedic Surgery Randers Regional Hospital Randers Denmark; ^4^ Department of Obstetrics and Gynecology Odense University Hospital Odense Denmark; ^5^ Department of Clinical Research University of Southern Denmark Odense Denmark

**Keywords:** cancer prevention, epidemiology and prevention, gynecological oncology, screening

## Abstract

The incidence and mortality of cervical cancer are high in Danish women aged 60 years and older who are about to exit the cervical cancer screening program. The present study aimed to describe the screening history in women ≥60 years old, diagnosed with cervical cancer in Denmark, 2009‐2013. We retrieved information on cases of cervical cancer and previous cervical cancer screening from national registries. During the study period, a total of 1907 women were diagnosed with cervical cancer, 574 (30.1%) of which were ≥60 years old. The majority of women were diagnosed with squamous cell carcinoma (73.7%) and advanced‐stage disease (ASD, ie, ≥FIGO IIB; 63.1%). The proportion of ASD increased with age, from 51.9% in women aged 60‐64% to 76.7% in women aged 75‐79. Among screened women (n = 377), 22.8% had a cervical cytology within 5 years of diagnosis, 73.3% of which were normal, and 45.1% were diagnosed with ASD. Women who had been sufficiently screened prior to screening exit (≥2 normal cytology test in the age interval 50‐59) accounted for 18.1%. Of note, 53.8% of the sufficiently screened women were diagnosed with ASD. Sufficiently screened women were less likely to be diagnosed with ASD compared to never‐screened women (53.8% vs 67.5%, *P < *0.020), but no difference was observed between sufficiently and insufficiently screened women (53.8% vs 63.4%, *P* = 0.091). Our findings suggest that cancer in older women may occur due to insufficient screening prior to screening exit, a low sensitivity of screening, and premature screening exit.

## 1 INTRODUCTION

Since the implementation of cervical cancer screening, cervical cancer incidence and mortality have declined significantly in developed countries, including Denmark.^1^ However, despite that screening is organized and free of charge for Danish women in the targeted age, the cervical cancer incidence and mortality rates in Denmark remain higher than the rates in other North European countries.^2^ The incidence is particularly high in Danish women aged 70‐75,^3^ for whom routine screening is no longer recommended.^4^ In 2016, nearly one‐third of cervical cancer cases in Denmark were found in women aged 60 and older, and their mortality rate was four times higher than for women <60.^2^, ^5^ To reverse this trend, it is important to understand whether these cancers occur because of insufficient or no screening, a lack of follow‐up, or a failure in screening. For years, there has been an ongoing debate on when to stop cervical cancer screening, as opinions on the value of screening older women have been divided. The International Agency for Research on Cancer (IARC) recommends to stop screening at age 60 or 65 if a woman has had at least two normal cervical cytology tests prior to the screening exit.^6^ American guidelines recommend cessation of screening at age 65 years in sufficiently screened women, defined as having had three consecutive normal cervical cytology tests or two consecutive normal cotests within 10 years of screening exit and no diagnosis of cervical intraepithelial neoplasia grade 2 or worse within the past 20 years.^7^ The decision on when to stop screening in developed countries is largely based on expert opinion and modeling studies, as research with a more rigorous methodology is limited.

Using recent data from nationwide registries, the present study aimed to describe the screening history in women ≥60 years old, diagnosed with cervical cancer in Denmark, 2009‐2013. More specifically, this study aimed to describe the overall screening history, the screening history 10 years prior to screening exit with respect to the IARC recommendations, and the potential impact of screening status on the stage of cervical cancer.

## 2 MATERIALS AND METHODS

Cervical cancer screening in Denmark is organized and free of charge, meaning that all female citizens in Denmark between 23 and 65 years of age receive a personal invitation letter every 3‐5 years after their last normal cytology, unless a woman has declined to participate. Cervical cancer screening began as opportunistic screening in the Copenhagen area in the mid‐1960s. The first national guidelines on cervical cancer screening were published in 1986, recommending all women aged 23‐59 years to undergo screening by cervical cytology every 3 years. New guidelines were published in 2007, recommending postponing screening exit to age 65. Furthermore, the screening interval was changed from every 3 years to every 5 years in women aged 50‐64, and a woman was recommended to exit the screening program at age 65 if the previous two cervical cytology tests were normal. The 2012 guidelines recommended to replace primary screening by cytology with an HPV test for women aged 60‐64 and to exit all women who tested HPV‐negative, regardless of previous screening history^4^; however, these changes were not implemented in all counties until August 2014. In Denmark, screening coverage was 73.5% in 2016.^8^ Only 11% of non‐participants in Denmark have deliberately opted out of the cervical cancer screening program.^8^

We conducted a nationwide population‐based cohort study in Denmark, where all citizens have access to comprehensive medical care at general practitioners and public hospitals free of charge due to a tax‐based health care system. Linkage of individual data from nationwide registries is possible due to the civil registration number (CPR), a unique code assigned to all citizens at birth or upon immigration. The present study included women ≥60 years old, diagnosed with cervical cancer in Denmark, from 1 January 2009 to 31 December 2013. Data on cervical cancer cases were collected from the Danish Cancer Registry (DCR) using the International Classification of Diseases, 10th revision (ICD‐10) codes: DC539 and DC53*. The DCR holds the CPR number for each individual, the diagnosis according to International Classification of Diseases, 7th revision (ICD‐7) or ICD‐10, date of diagnosis, tumor location, tumor histology according to the International Classification of Diseases for Oncology, 3rd edition, and tumor, node, metastasis (TNM) classification as defined by the American Joint Committee of Cancer. In the present study, the TNM classification was converted to the International Federation of Gynecology and Obstetrics (FIGO) classification following the existing guideline.^9^ We classified ≤FIGO IIA as early‐stage disease (ESD) and ≥FIGO IIB as advanced‐stage disease (ASD).

Information on previous screening history was obtained from the Danish National Pathology Registry (DNPR). The DNPR was established in 1997 and holds information on pathology specimens from all pathology departments in Denmark. Data are transferred from the Danish Pathology Databank, which is updated on a daily basis through linkage to all pathology departments in Denmark. Most pathology departments have transferred data on specimens obtained during 1978‐1998; however, data collected prior to 1998 are considered incomplete. All diagnoses in the Pathology Registry are classified according to SNOMED (Systematized Nomenclature of Medicine), a system consisting of six code strings. We extracted information on the dates and results of all previous cervical cytology tests and histology (ie, cervical punch biopsies, cervical curettage, and cone biopsies). We classified cytology tests as follows: inadequate, normal, low‐grade disease, high‐grade disease, and cancer. A complete list summarizing the classification of cervical cytology results is provided in Table S1.

### 2.1 Statistics

Ever‐screened women were defined as women with at least one recorded cervical cytology test in the DNPR, up to 6 months prior to cervical cancer diagnosis. Conversely, never‐screened women were defined as women with no record of cervical cytology. We excluded cervical cytology tests obtained within 6 months of cervical cancer diagnosis, as these smears were considered diagnostic. To describe recent screening history, we categorized ever‐screened women in four groups: women with at least one record of a cervical cytology test within 0.5‐5 years of diagnosis, women with no record of a cervical cytology within 5 years of diagnosis but with a record of at least one cervical cytology test within 5‐10 years of diagnosis, women with no record of a cervical cytology within 10 years of diagnosis but with a record of at least one cervical cytology test within 10‐15 years of diagnosis, and lastly women with no record of a cervical cytology test within 15 years of diagnosis but with at least one record of cervical cytology test more than 15 years prior to diagnosis.

To describe screening history with respect to the IARC recommendations, we conducted a stratified analysis of ever‐screened women by categorizing them based on their screening history in the 10‐year period prior to screening exit (ie, age 50‐59). Sufficiently screened women were defined as women who had a record in the DNPR of at least two normal cervical cytology tests with at least a 3‐year interval in the age interval 50‐59 and with no record of an abnormal cervical cytology in this age interval. Insufficiently screened women included women without a record of a cervical cytology test in the age interval 50‐59 and women with one or more records of a cervical cytology but not complying with the restrictions mentioned above (eg, abnormal tests results and cervical cytology tests taken <3 years apart). Never‐screened women were categorized separately because we wanted to explore potential differences among never‐screened and insufficiently screened women. Although women in the present study were diagnosed during 2009‐2013, and thus after screening exit was postponed from age 59 to 64 (ie, 2007), we decided to include women aged 60 years and older because the majority of cases were likely screened following the screening recommendations prior to 2007 and would have exited the screening program at 59 years of age.

All categorical data were analyzed using the chi‐square test. Proportion analysis was used to calculate the 95% confidence intervals for proportions, and a Wilcoxon‐Mann‐Whitney test was used to analyze nonparametric data. A *P*‐value <0.05 was considered statistically significant. Statistical analyses were conducted using STATA 14 (StataCorp LP, College Station, TX). The study was approved by the Danish Data Protection Agency (1‐16‐02‐295‐15). According to Danish Legislation, it is not required to obtain ethics approval for registry‐based research studies.

## 3 RESULTS

**Figure 1:**
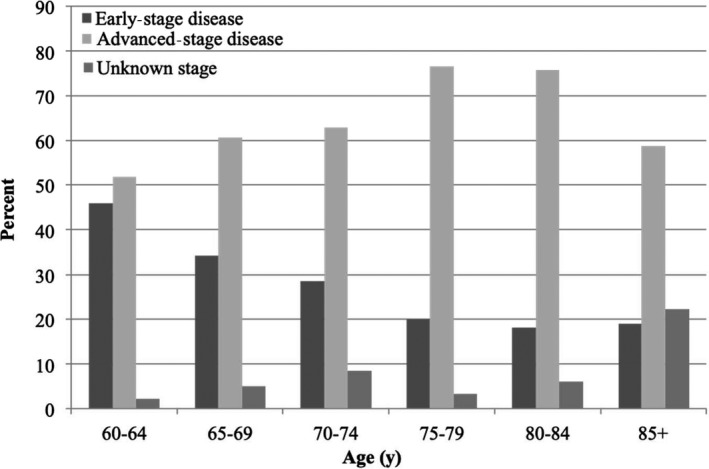
Distribution of early‐stage disease (ESD), advanced‐stage disease (ASD), and unknown stage by age

**Figure 2:**
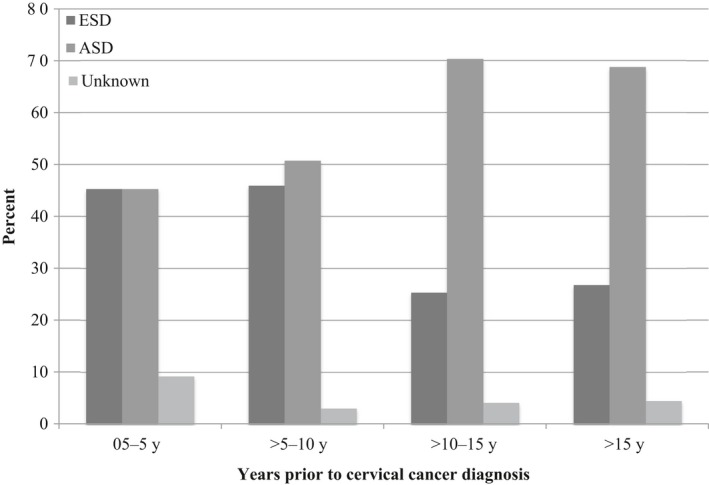
Stage of disease in women undergoing screening within 0.5‐5, >5‐10, >10‐15, and >15 y of cervical cancer diagnosis. ASD, advanced‐stage disease; ESD, early‐stage disease

During the study years 2009‐2013, a total of 1907 women were diagnosed with cervical cancer in Denmark, 574 women (30.1%) of which were aged 60 or older and included in the present study. Basic characteristics of the study population are summarized in Table 1. The median age of the study population was 72 years (interquartile range [IQR] 68‐83). Of all women, 73.7% (n = 423) were diagnosed with squamous cell carcinoma (SCC), and 63.1% (n = 362) were diagnosed with ASD. As illustrated in Figures 1 and 2, the proportion of women diagnosed with ASD varied by age and time since last cytology. The proportion was lowest in women aged 60‐64 (51.9%) and highest in women aged 75‐79 (76.7%), and the proportion increased from 45.3% in women with cytology within 5 years of diagnosis to 68.8% in women whose most recent cytology was more than 15 years prior to diagnosis.

**Table 1:** Characteristics of women aged 60 y and older diagnosed with cervical cancer during 2009‐2013

Variables	Study subjects (n = 574)		
	n	%	95% CI
Age at diagnosis			
60‐64 y	133	23.2	19.9; 26.8
65‐69 y	117	20.4	17.3; 23.9
70‐74 y	105	18.3	15.3; 21.7
75‐79 y	90	15.7	12.9; 18.9
80‐84 y	66	11.5	9.1; 14.4
≥85 y	63	11.0	8.7; 13.8
Year of diagnosis			
2009	129	22.5	19.2; 26.1
2010	98	17.1	14.2; 20.4
2011	117	20.4	17.3; 23.9
2012	112	19.5	16.5; 23.0
2013	118	20.6	17.4; 24.1
Histology			
Squamous cell carcinoma	423	73.7	69.9; 77.1
Adenocarcinoma	86	15.0	12.3; 18.2
Adenosquamous carcinoma	18	3.1	2.0; 4.9
Other carcinoma	47	8.2	6.2; 10.7
Stage of disease			
Early‐stage disease (FIGO≤IIA)	173	30.1	26.5; 34.0
Advanced‐stage disease (≥FIGO IIB)	362	63.1	59.0; 66.9
Unknown stage	39	6.8	5.0; 9.2
History of abnormal cervical cytology			
Low‐grade disease	93	16.2	13.4; 19.5
High‐grade disease^a^	67	11.7	9.3; 14.6
History of surgical treatment			
Cone biopsy/LEEP^b^	38	6.6	4.8; 9.0

a Includes smears revealing cancer.

b Loop electrosurgical procedure.

The proportion of ever‐screened women was 65.7% (n = 377). Ever‐screened women were significantly younger than never‐screened women (*P* < 0.001), and more likely to be diagnosed with adenocarcinoma (*P < *0.019) and early‐stage disease (*P* < 0.006; data not shown). The median time from the last cervical cytology test to cervical cancer diagnosis was 13.3 years (95% CI 11.5‐14.3) in the overall population, and 8.4 years (95% CI 5.7‐12.2) and 14.6 years (95% CI 13.5‐15.6) in women with ESD and ASD, respectively (Table 2). When stratified by 5‐year age‐groups, the median time from last cytology test to cervical cancer diagnosis increased steadily, from 5.9 years in women aged 60‐64 years to 19.1 years in women aged 85 years and older. Of note, 60.4% of never‐screened women were born before 1943, meaning that they may have been too old to be screened when screening became organized and data in the pathology registry were complete (ie, 1998).

**Table 2:** Patient and tumor characteristics of the study population according to the screening history in the age interval 50‐59 y

	Sufficiently screened women^a^ (n = 104)		Insufficiently screened women^b^ (n = 273)			Never‐screened women^c^ (n = 197)		
	Years	95% CI	Years	95% CI	*P*‐value^d^	Years	95% CI	*P*‐value^d^
Median age at diagnosis	68	67; 71	69	68; 72	0.075	76	74; 77	<0.001
Median time since last cytology								
All stages	7.4	5.8; 9.9	15.1	14.0; 16.8	<0.001	—	—	—
Early‐stage disease (≤FIGO IIA)	5.8	5.1; 9.5	12.5	6.1; 15.5	0.041	—	—	—
Advanced‐stage disease (≥FIGO IIB)	9.7	6.9; 11.3	16.3	14.6; 18.5	<0.001	—	—	—

	n (%)	95% CI	n (%)	95% CI	*P*‐value^d^	n (%)	95% CI	*P*‐value^d^
Histology								
Squamous cell carcinoma	60 (57.7)	47.9; 66.9	202 (74.0)	68.4; 78.9	0.002	161 (81.7)	75.6; 86.6	<0.001
Adenocarcinoma	24 (23.1)	15.9; 32.3	42 (15.4)	11.6; 20.2	0.079	20 (10.2)	6.6; 15.3	0.003
Adenosquamous carcinoma	7 (6.7)	3.2; 13.6	7 (2.6)	1.2; 5.3	0.056	4 (2.0)	0.8; 5.3	0.039
Other carcinoma	13 (12.5)	7.3; 20.5	22 (8.1)	5.4; 12.0	0.185	12 (6.1)	3.5; 10.5	0.056
Stage of disease								
Early‐stage disease (≤FIGO IIA)	42 (40.4)	31.3; 50.2	86 (31.5)	26.2; 37.3	0.104	45 (22.8)	17.5; 29.3	0.001
Advanced‐stage disease (≥FIGO IIB)	56 (53.8)	44.1; 63.3	173 (63.4)	57.5; 68.9	0.091	133 (67.5)	60.6; 73.7	0.020
Unknown stage	6 (12.5)	7.3; 20.5	14 (8.1)	5.4; 12.0	0.804	19 (6.1)	3.5; 10.5	0.247

a Women with a record of at least two normal cervical cytology tests in the age interval 50‐59 y taken at least 3 y apart.

b Women with at least one record of a cervical cytology test at some point in their life, but not compliant with the criteria for sufficient screening.

c Women with no record of a cervical cytology.

d Compared to sufficiently screened women.

**Figure 3:**
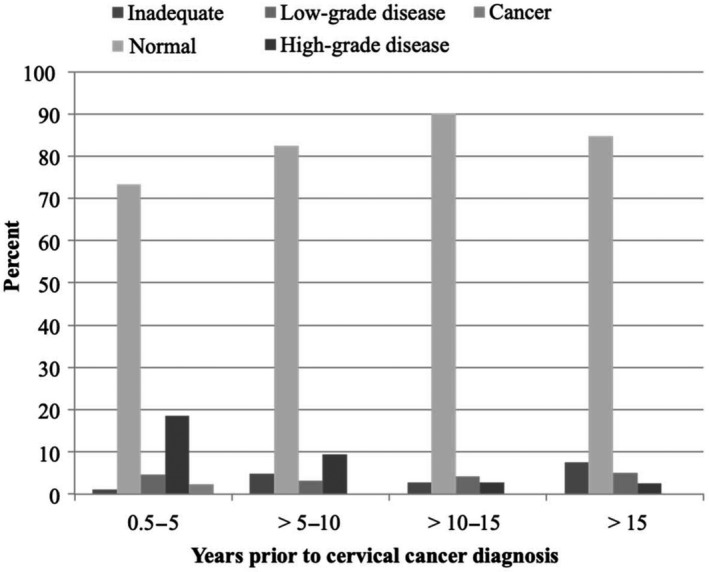
Results of cervical cytology tests obtained prior to a diagnosis of cervical cancer in 377 ever‐screened women

Among ever‐screened women (n = 377), the proportion of women screened 0.5‐5 years, >5‐10 years, >10‐15 years, and >15 years prior to cervical cancer diagnosis was 22.8% (n = 86), 16.7% (n = 63), 18.8% (n = 71), and 41.6% (n = 157), respectively (data not shown). The majority of cervical cytology tests taken within these time periods were normal, ranging from 73.3% to 90.1% (Figure 3). Of note, 73.3% (95% CI 62.7‐81.7) of cervical cytology tests obtained within 5 years of cervical cancer diagnosis were normal, whereas 18.6% (95% CI 11.6‐28.5) and 2.3% (95% CI 0.6‐9.1) of cervical cytology tests revealed high‐grade disease and cancer, respectively. Among women with a record of screening 5‐10 years prior to diagnosis, nearly 10% of cervical cytology tests revealed high‐grade disease.

Among all women with cervical cancer, 18.1% (n = 104) had been sufficiently screened in the age interval 50‐59 and were eligible to exit the screening program according to the IARC recommendations and the 2007 guidelines in Denmark, and 75 women (13.1%) had records of normal cervical cytology results exclusively. Conversely, 273 women (47.6%) had an insufficient screening history in the age interval 50‐59 and were therefore not eligible to exit the screening program. Of the insufficiently screened women, 40 women (7.0%) had a record of at least two cervical cytology tests within a 3‐year interval where at least one test was abnormal. Of note, 120 women (20.9%) had no record of a cervical cytology in the age interval 50‐59 years, of whom 29 women (24.2%) were born since 1947, and the data on their screening histories since age 50 are considered complete.

Table 2 displays the characteristics of sufficiently screened, insufficiently screened, and never‐screened women. Women who were sufficiently screened had a significantly lower risk of being diagnosed with squamous cell carcinoma compared to insufficiently screened (57.7% vs 74.0%; *P < *0.002) and never‐screened women (57.7% vs 81.7%; *P* < 0.001). Additionally, sufficiently screened women were significantly less likely to be diagnosed with ASD compared to never‐screened women (53.8% vs 67.5%, *P* < 0.020). Insufficiently screened women were more likely to be diagnosed with ASD compared to sufficiently screened women; however, this finding was not statistically significant (53.8% vs 63.4%, *P* = 0.091). Among sufficiently screened women, the median time from last cervical cytology to cervical cancer diagnosis was 5.8 years in women with ESD and 9.7 years in women with ASD. In contrast, for women who had been insufficiently screened, the median time from last cervical cytology to cervical cancer diagnosis was 12.5 years in women with ESD and 16.3 years in women with ASD (Table 2).

## 4 DISCUSSION

In the present study, two‐thirds of women diagnosed with cervical cancer at age 60 years and older had at least one record of screening prior to their diagnosis. Among ever‐screened women, 22.8% (n = 86) had cervical cytology recorded within 0.5‐5 years prior to diagnosis, and the majority of these smears were normal (73.7%), whereas 20.9% revealed high‐grade disease or worse. The median time since last cytology increased with age, as did the proportion of women diagnosed with ASD. Of all women with cervical cancer, 18.2% (n = 104) had been sufficiently screened and were eligible to stop screening according to the IARC recommendations. Sufficiently screened women were less likely to be diagnosed with ASD compared to never‐screened women (*P < *0.020). However, there was no significant difference in the proportion of ASD between sufficiently and insufficiently screened women (*P* = 0.091).

Similar to previous studies, a large proportion (34.3%) of cervical cancer cases among women ≥60 years old were found in women who had never been screened.^10^ This is worrisome, as regular screening has been shown to reduce cervical cancer incidence and mortality by 82% and 92%, respectively.^11^, ^12^ However, the high proportion of never‐screened women observed in the present study may be due to the inclusion criteria being women diagnosed aged 60 and older, since they may have lived in a period where organized screening was not available or they may have been too old to be invited when organized screening was implemented (ie, a period effect of screening).^13^ When comparing our results with previous studies, it is therefore important to consider that the proportion of cervical cancer cases with no record of screening varies significantly by calendar time, age, and across countries. These variations may reflect the period effect of screening, including differences in screening policy across countries, changes in screening recommendations over time, and cohort effects.^13^ In the United States, where screening is opportunistic, the proportion of cervical cancer cases aged 16‐90 years old with no previous screening has been reported to be as low as 2.2%‐7%,^14^, ^15^ with an increase in the proportion of unscreened or insufficiently screened women with increasing age.^16^ In Europe, studies have reported that 25%‐40% of cervical cancer cases have never been screened prior to their diagnosis,^17^, ^18^, ^19^ and similarly to the United States, older women are reportedly less likely to have been screened compared to younger women^19^, ^20^; however, this trend has been reported to decline over calendar time, as more women may have had the opportunity to be screened.^13^

The incidence and mortality rates of cervical cancer are high in older Danish women.^2^, ^3^ It is critical to understand why these cancer cases occur in order to design the interventions needed to reduce the disease rates. The fact that only 22.8% of ever‐screened women had a screening record 0.5‐5 years prior to diagnosis does not necessarily imply non‐compliance with the current screening guidelines, but rather that the majority of these women were likely no longer to be invited to participate in the screening program due to their age. Similar to previous studies,^13^, ^21^ our results suggest a reduced sensitivity of screening with cytology in older women, as 73% of cervical cytology tests obtained within 0.5‐5 years of diagnosis were normal. The lower test sensitivity may be due to aspects of specimen retrieval such as vaginal atrophy, which causes lower cellularity of the specimen, an insufficient sample of the transformation zone because it retracts into the cervical canal as a woman ages, or a result of false interpretation of the specimen. In the study by Mowack et al,^21^ the latter explanation seemed to account for a small fraction only. Nevertheless, in Denmark, an audit is now being conducted in all incident cervical cancer cases with a record of a cervical cytology within the preceding three (23‐ to 49‐year‐olds) to 5 years (50‐ to 64‐year‐olds). The fact that the proportion of adenocarcinoma was higher in sufficiently screened women compared to insufficiently and never‐screened women is in line with previous studies in which screening with cytology has been reported to reduce the risk of advanced stage but not early‐stage adenocarcinoma.^22^ Other studies have reported an increasing proportion of adenocarcinoma over calendar time in a screened population,^23^ and in younger Danish women, the incidence of adenocarcinoma is reportedly rising.^24^

The increase in the proportion of women with ASD with time since last cytology is also in line with a previous study in which regular screening with cytology was shown to significantly reduce the risk of ASD, regardless of age.^11^ The increase in ASD with age in the current study parallels the observed increase in the median time since last cytology with age, as these women will have exited screening at age 60. This may suggest that continuing screening beyond the age of 64 would reduce the risk of ASD through earlier detection.

Cervical cancer screening guidelines from the United States, Denmark, and the IARC state that it is safe to stop cervical cancer screening at age 65,^4^, ^7^, ^25^ as the risk of cervical cancer is considered very low in sufficiently screened women (ie, 2 [IARC] or 3 [United States] consecutive normal cervical cytology results or 2 consecutive cotests [United States]) in the 10‐year period prior to screening exit. The decision on when to recommend cessation of screening was mainly based on modeling studies and expert opinion.^7^, ^25^ Since then, Castanon et al have reported that women with an adequate negative screening history, defined as three normal cervical cytology tests in the age interval 50‐64 years, have a 75% lower risk of cervical cancer occurring after 65 years of age compared to women who have not been screened in the same age interval.^11^, ^26^ However, this reduction in risk was reported to decline over time,^26^, ^27^ possibly ending 5‐7 years after the latest normal cytology test,^28^ which may suggest that continuing screening after age 65 may be important in order to reduce the risk of cervical cancer and death from the disease.^26^, ^28^, ^29^ In the present study, we were unable to explore the impact of sufficient screening on risk of cancer after screening exit, as we did not include controls.

It has been estimated that approximately 50% of cervical cancers may be attributed to a lack of screening.^10^ Dinkelspiel et al^30^ reported that the majority of cancers (75%) occurring after the age of 65 years may be attributed to an insufficient screening history in the 10‐year period preceding screening exit or due to no screening attendance at all. In the present study, one out of five women who were diagnosed with cervical cancer at age 60 or older had two normal cervical cytology results in the age interval 50‐59 years and were eligible to exit screening according to the IARC and the Danish recommendations from 2007. Results from the US study and the present study may suggest that 75%‐80% of cancer cases occurring after screening exit might have been avoided had these women been sufficiently screened and that attention should be directed toward interventions aiming at increasing the participation rate and improving the follow‐up after an abnormal cytology. However, in the present study, 53.8% of the women diagnosed with cancer at age 60 years or older who had been sufficiently screened in the age interval 50‐59 years were diagnosed with ASD. Among sufficiently screened women, the proportion of women with ASD increased with time since last cytology, suggesting that continuing screening may reduce the risk of ASD. The high proportion of ASD among sufficiently screened women and overall is worrisome, particularly because ASD is associated with more aggressive treatment, a higher morbidity, and because 5‐year survival rate is significantly lower in ASD (64%) compared to ESD (>90%).^31^

According to the current Danish cervical cancer screening guidelines (2012), women aged 60‐64 should undergo primary HPV screening, and a woman can stop screening at age 65 years if the HPV test is negative, regardless of previous screening history.^4^ Although a recent modeling study has reported a low lifetime risk of cervical cancer after an HPV‐negative test at age 55,^27^ the “true” risk of cervical precancer and cancer after one negative HPV test remains unclear, as empirical studies with sufficient follow‐up time are limited. However, a Dutch observational study reported an increased risk of CIN2+ (cervical intraepithelial neoplasia grade 2 or worse) in HPV‐negative women who had previously had an HPV infection,^32^ suggesting that a previous HPV‐positive test result may confer an increased risk of CIN2+ later in life. Continued surveillance will be critical to evaluate the potential impact of the HPV exit test on risk of cervical precancer and cancer in Danish women ≥65 in future studies, especially because conflicting results have been reported on HPV‐based screening. Ronco and Gyllensten reported a higher detection rate of cervical precancers when using HPV‐based screening,^33^ including in postmenopausal women,^34^ while other studies report suboptimal sensitivity for cervical intraepithelial neoplasia grade 3 or worse (CIN3+).^35^ Another study reported that 19% of cervical cancers were missed by primary HPV screening.^36^ This could be due to a low sensitivity of colposcopy in older women^37^, ^38^ or because the proportion of HPV‐negative cancer is substantially higher in older women compared to younger women.^39^, ^40^, ^41^

This study has some limitations that must be addressed. Firstly, although Denmark has one of the oldest and most complete pathology registries in the world, data prior to 1998 are considered incomplete. We cannot rule out that some of the oldest women in our study population may have been screened prior to 1998, which could bias the results toward a higher proportion of ever‐screened women. Secondly, we were unable to adjust for other covariates known to be associated with screening attendance, such as socio‐economic status. Thirdly, the lack of a statistically significant difference between insufficiently and sufficiently screened women may be due to a small sample size. Strengths include the use of high‐quality national registries instead of relying on self‐reported screening history.

In conclusion, given that 80% of cervical cancer cases among women ≥60 occurred in insufficiently or never‐screened women, and because previous studies report a lower participation rate in screening among women aged 50‐64 compared to younger women,^42^, ^43^ more attention should be given to interventions aimed at increasing the participation rate in screening among women aged 50‐64. An increase in screening participation during the age interval 50‐64 years may likely reduce the cervical cancer incidence in women ≥65. However, it is worrisome that one out of five women in the present study developed cervical cancer despite meeting the exit criteria. Importantly, 53.8% of these women were diagnosed with ASD, which is known to be associated with a lower survival rate. The fact that the proportion of ASD increased with time since last cytology and by age may suggest that continuing screening beyond the age of 65 may decrease the risk of ASD, which may subsequently lead to a decline in the cervical cancer mortality rate. Future studies should explore the impact of the HPV exit test on cervical cancer rates in Denmark.

## CONFLICT OF INTEREST

None declared.
