# Supplementary material for: Cervical cancer screening history prior to a diagnosis of cervical cancer in Danish women aged 60 years and older—A national cohort study
Source: Cancer Med. 2019 Jan 1;8(1):418–27. doi: 10.1002/cam4.1926 (PMC6346234; doi:10.1002/cam4.1926)
Supplement: Supplementary file 1 [file CAM4-8-418-s001.docx]

Supplementary Table 1. Classification of cervical cytology tests

| **Cytology classification** | **Cytology diagnoses in the Danish National Pathology Registry** |
| --- | --- |
| Inadequate | Not representative, not sufficient material for diagnostic assessment, material with insufficient amount of endocervical cells, not suitable for diagnostic assessment, hemorrhage |
| Normal | Normal, inflammation (acute and chronic), inflammation-related cell changes, reactive/reparative cellular changes, abnormal cyto-hormonal pattern, metaplasia, atrophy, parakeratosis, dyskeratosis, hyperkeratosis |
| Low-grade disease | Atypia, ASC-US (atypical squamous cells of undetermined significance), koilocytosis, LSIL (low grade squamous intraepithelial lesion), low-grade dysplasia, dysplasia not specified |
| High-grade disease | HSIL (high grade squamous intraepithelial lesion), ASC-H (atypical squamous cells- cannot exclude HSIL), AIS (adenocarcinoma in situ), CIS (cervical carcinoma in situ), cells suspicious of malignancy, suspicious of adenocarcinoma, moderate dysplasia, severe dysplasia, metaplastic dysplasia |
| Cancer | Squamous cell carcinoma, adenocarcinoma, micro-invasive squamous cell carcinoma, carcinoma, adenosquamous carcinoma, tumor cells, malignant tumor cells |
